# Understanding Users’ Acceptance of Artificial Intelligence Applications: A Literature Review

**DOI:** 10.3390/bs14080671

**Published:** 2024-08-02

**Authors:** Pengtao Jiang, Wanshu Niu, Qiaoli Wang, Ruizhi Yuan, Keyu Chen

**Affiliations:** 1 School of Information Science and Engineering, NingboTech University, Ningbo 315100, China; pengtao.jiang@nottingham.edu.cn; 2 Nottingham University Business School China, University of Nottingham Ningbo China, Ningbo 315100, China; russa.yuan@nottingham.edu.cn; 3 Business School, Ningbo University, Ningbo 315211, China; niuwanshu@nbu.edu.cn; 4 School of Management, Zhejiang University, Hangzhou 310058, China; towangqiaoli@163.com

**Keywords:** AI, user acceptance, AI service provider, AI task substitute, systematic literature review

## Abstract

In recent years, with the continuous expansion of artificial intelligence (AI) application forms and fields, users’ acceptance of AI applications has attracted increasing attention from scholars and business practitioners. Although extant studies have extensively explored user acceptance of different AI applications, there is still a lack of understanding of the roles played by different AI applications in human–AI interaction, which may limit the understanding of inconsistent findings about user acceptance of AI. This study addresses this issue by conducting a systematic literature review on AI acceptance research in leading journals of Information Systems and Marketing disciplines from 2020 to 2023. Based on a review of 80 papers, this study made contributions by (i) providing an overview of methodologies and theoretical frameworks utilized in AI acceptance research; (ii) summarizing the key factors, potential mechanisms, and theorization of users’ acceptance response to AI service providers and AI task substitutes, respectively; and (iii) proposing opinions on the limitations of extant research and providing guidance for future research.

## 1. Introduction

The rapid development of artificial intelligence (AI) has provided rich opportunities for industrial development and social progress. With an expectation of shaping commercial value, improving versatility, and promoting efficiency, AI technology is now increasingly applied into online retailing [1], customer service [2,3], digital innovation [4,5], and providing management support [6,7,8]. Industry reports show that the scale of China’s artificial intelligence industry reached CNY 195.8 billion in 2022, and it is expected to reach CNY 612.2 billion by 2027 [9]. It is worth noting that the success of AI implementation relies on not only technological progress, but also users’ acceptance [10,11]. Although technology implementers place high expectations for AI to improve user experience and performance, the application of AI in multiple fields has reported low actual usage rates [12,13]. Thus, it is vital to understand users’ reactions to AI applications and analyze factors that are relevant to user’s acceptance behavior [2,14].

Artificial intelligence (AI) is “the frontier of computational advancements that references human intelligence in addressing ever more complex decision-making problems” [15]. AI applications are thus “able to perform tasks that require cognition and were formerly typically associated with humans” [16]. Numerous studies have examined user acceptance of AI applications, but revealed mixed results. For example, You, et al. [17] found that users appreciated algorithmic advices more than human advices. In contrast, Longoni, et al. [18] indicated that users were reluctant to use medical AI both in hypothetical or real choices. Furthermore, Garvey, et al. [19] showed that users accepted AI agent more when receiving bad messages, but responded more positively to human agent when receiving good news. Meanwhile, although a large body of research on AI acceptance has focused on various AI application form (e.g., chatbots, AI-based decision-making systems, AI-embedded smart home devices, and autonomous vehicles), there is no consensus on what distinguishes these forms from others and what roles these AI applications plays in human–AI interactions. However, differences in users’ attribution, perception, and acceptant criteria exist among these different AI application forms. For instance, research has indicated that user acceptance of an AI system, which is designed to deliver improved services, is associated with perceptions of algorithmic credibility, usefulness, convenience, and trust [20], while users’ attitudes toward AI systems, which replace users in completing tasks, may result from perceived threat, performance risk, and inertia [21]. This indicates that some differences are present between different forms of AI applications related to user acceptance, in particular, in whether users treat AI as service provider or task substitute. Accordingly, we categorize AI applications into AI service providers and AI task substitutes. Specifically, AI service provider refers to an AI application that provides services to users instead of a human agent or ordinary product [17,18,19], such as AI providing shopping recommendations, customer service, and advising services. AI task substitutes are AI applications that replace users to complete certain tasks [18,22,23], such as AI-generated diagnostic systems, AI-based substitutive decision-making systems, and AI teammates. Decades of experience with new technology implementation suggest that the role of the technology is an important determinant of user acceptance [24,25]. However, despite this, very few attempts have been made to synthesize the extant research in the current works.

To fill this gap, this study aims to analyze the literature regarding users’ acceptant attitudes to AI service providers and task substitutes. A comprehensive review of user’s acceptance of AI applications can help identify collective knowledge of the extant literature, improve understanding of the mixed findings, and provide guidance for future investigations of this important and relevant issue of AI technology implementation. There are several published literature reviews about AI on organizational strategy [16], business value [26], and future of work [27]. To the best of our knowledge, this study differs from the prior review research on AI implication by providing a systematic literature review on AI acceptance from the end user’s perspective rather than focusing on the objectives of technology implementers. Additionally, prior work did not explicitly discern the roles of AI (e.g., AI service providers and task substitutes). In this article, we (i) provide an overview of the exiting methodologies and theoretical frameworks used to investigate AI acceptance; (ii) synthesize key factors, potential mechanisms, and theorizing logics underlying users’ AI acceptant responses to AI service providers and task substitutes, respectively; and (iii) propose opportunities for future research.

The paper is organized as follows. The process of literature identification and selection is firstly explained in Section 2. The journal distribution, methodology overview, and outcome variables of the reviewed studies are also presented. Section 3 and Section 4 analyze users’ different attitudes toward AI service providers and AI task substitutes, respectively. Section 5 summarizes the theoretical frameworks used in our reviewed papers. Finally, gaps and limitations of the extant literature, future research directions, and limitations of the present paper are discussed. This paper ends with a conclusion.

## 2. Methods

**Figure 1:**
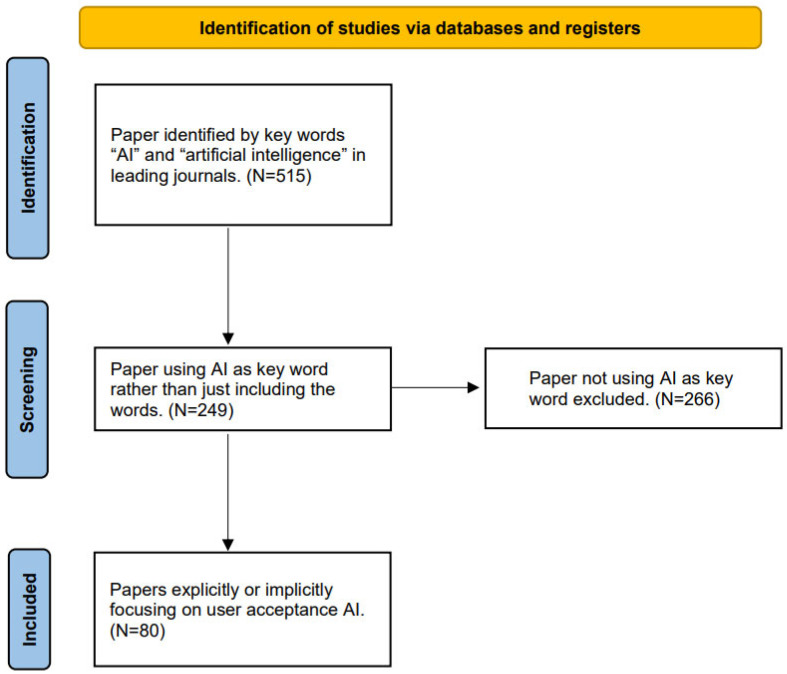
PRISMA flowchart.

The flowchart based on the PRISMA guidelines (Figure 1) illustrates the process of searching, screening, and ultimately selecting articles for this study. The final selection includes articles from 12 leading journals, organized into nine major research methods. Furthermore, this chapter lists the types of outcome variables concerning users’ acceptance of AI service providers.

### 2.1. Literature Identification and Selection

Given the huge volume and variety of AI research, our search process was restricted to the newest papers published between 2020 and 2023, and was conducted in leading journals in marketing, information systems and behavioral science domains, including Management Science, Marketing Science, MIS Quarterly, Information Systems Research, Journal of Marketing, Journal of Marketing Research, Journal of Consumer Research, Journal of the Association for Information Systems, Journal of Management Information Systems, International Journal of Information Management, Information & Management, Computers in Human Behavior, and Decision Support Systems. These journals were selected due to their outstanding contributions to technology-acceptance-related knowledge [28]. Specifically, our selection of “Computers in Human Behavior”, a leading journal in the behavioral sciences, is well-regarded for its authoritative contributions, high impact, and relevance to our study’s focus on technology acceptance. Thus, our selection adequately covers significant contributions from both the behavioral sciences and information systems domains.

To ensure no relevant paper was missed, the process began with an automated search with key words “AI” and “artificial intelligence” in the journals mentioned above. After excluding the repeated papers, this process identified 515 articles. Then, we restricted the search to papers using “AI” or “artificial intelligence” as key words and excluded studies that just mentioned AI. This process brought up 249 papers. A manual search was then conducted to ensure that only papers related to user acceptance were included. The key words used in this stage were quite diverse because of the variety of conceptualization of user acceptance in these papers. Thus, we read the abstracts and other related contents of all papers to identify studies that either explicitly or implicitly focus on user acceptance of AI. Finally, 80 papers were included in our analysis. The PRISMA flow diagram summarizes the literature selection process (Figure 1).

### 2.2. Overview of Reviewed Studies

Table 1 summarizes the journal distribution and methodology overview of the reviewed studies. Sixteen publications were published in UTD24 journals. Most studies were published in Computers in Human Behavior and International Journal of Information Management. Most of the studies used quantitative methodologies (63 papers), 6 papers adopted qualitative approaches to explore user acceptance of AI, and 11 papers combined multiple methods (e.g., empirical estimation and controlled behavioral experiments, survey, and lab experiments). Specifically, controlled behavioral experiments (29 papers) and survey (26 papers) were the two main approaches for user acceptance research. Only 5 papers conducted field experiments. Among qualitative studies, most research employed case studies (3 papers), 2 papers used interviews, and 1 paper conducted a two-year longitudinal study. Among the mixed-method studies, 6 papers combined qualitative methods and quantitative studies, 4 papers conducted a series of experiments and one survey, and 1 paper tested the proposed model by empirical estimation on real-world data and 4 controlled experiments (see in Table 2). Moreover, 1 paper used a game model to reveal how different expert accept AI tools.

### 2.3. Overview of Conceptualization

Regarding users’ acceptance of AI, the reviewed studies focused on a vast pool of outcome variables (see in Table 3). We categorized the outcome variables as behaviors, behavioral intentions, and perceptions. A total of 14 publications adopt users’ real behaviors to investigate how users accept an AI service provider or task substitute, including AI acceptance behavior (5 papers), AI usage behavior (6 papers), purchase behavior (2 papers), or user performance (1 paper) after AI acceptance. To analyze users’ real behaviors, these studies mainly relied on empirical estimation and field experiment. Most studies reported users’ behavioral intentions by means of survey and controlled behavioral experiments. For example, research has examined users’ intention to accept AI (18 papers), intention to use AI (23 papers), intention to purchase after AI acceptance (3 papers), and intention to self-disclosure to obtain better AI service (1 papers), as well as users’ tendency to perform better (4 paper) or resist AI (3 papers). Additionally, several studies also observed AI acceptance through the lens of user perception, such as attitude toward AI (6 papers), trust in AI (14 papers), and satisfaction with AI (6 papers).

## 3. Results of Literature Review on User Acceptance

In our review, we categorized the papers by role of AI, that is, AI service provider or AI task substitute. Two research assistants categorized and coded the 80 papers according to AI’s roles in human–AI interaction. When the categorization results coded by two research assistants were consistent, the categorization was adopted; when the categorization results coded by two research assistants were inconsistent, the final categorization results were determined after discussion. Finally, out of the 80 papers, 61 were classified as studies on user acceptance of AI service providers (see Table 4), while 19 were categorized under research on user acceptance of AI task substitutes (see Table 5).

### 3.1. Results of Literature Review on User Acceptance of AI Service Providers

Based on the 61 papers classified as studies on AI service providers, we summarize users’ acceptant responses to AI service providers and key findings of these research. Based on the definition, AI advisors, AI-based service agents, and other AI-based applications that benefit people in various areas were identified as AI service providers in our analysis. For example, AI advisors include judge–advisor systems, AI-based recommenders, medical AI applications, etc. Examples of AI-based service agents include AI marketing agents, customer service chatbots, and AI-based smart healthcare services. Moreover, other AI service providers include AI instructors for online learning, AI coaches for sales training, AI-embedded mixed reality for retail shopping, etc.

Despite the calls for AI implication for the future of society [100,101,102], our reviewed studies found mixed evidence showing that the links between AI service providers and high level of user acceptance were not always supported, or even showed a reversed result. Only 3 of the 61 papers reported a positive relationship between AI service providers and user acceptance. A total of 9 of the 61 papers provided evidence for users’ AI aversion responses. The majority of the reviewed studies (49 papers) reported conditional results.

Firstly, three studies showed experimental evidence for AI service provider appreciation. You, Yang and Li [17] found that users exhibit a strong algorithm appreciation. That is, people accept AI advice more than that generated by humans even when the prediction errors of AI algorithms have been acknowledged. Since people believe that an AI algorithm is able to give more accurate and reliable advice than humans, and they exhibit higher trust in AI-provided advice. From the perspective of responsibility attribution, Gill [29] revealed that harm to a pedestrian by an autonomous vehicle is more acceptable for users, due to the goal of self-protection to remove themselves from moral responsibility. Schanke, Burtch and Ray [2] observed that consumers are more willing to accept and self-disclose to a chatbot with anthropomorphic features (i.e., humor, communication delays, and social presence). Taken together, users tend to accept AI service providers because of the expectation that AI is more accurate, reliable, and able to take responsibility for the harm it causes. Consumers even increase sensitivity to offers provided by AI service providers due to a fairness evaluation or negotiating mindset. In our reviewed papers, advantages in accuracy, reliability, and responsibility are key factors that determine users’ appreciation for AI service providers; trust and satisfaction are the main mechanisms for forming positive user acceptance attitudes.

Secondly, nine papers observed a response of AI service provider aversion. A possible explanation is that people have doubts about the ability of artificial intelligence to understand human decision-making processes [36]. For example, Peng, van Doorn, Eggers, and Wieringa [30] found that consumers believed that AI is not competent in emotional support and, thus, they are reluctant to accept AI services for warmth-requiring tasks. Similarly, Luo, Tong, Fang, and Qu [32] observed that although chatbots perform as effectively as proficient workers, the disclosure of chatbot identity will reduce customer purchase rates. Mechanism exploration showed that consumers believe an AI-based servicer lacks knowledge and empathy. In the context of peer-to-peer lending, Ge, Zheng, Tian, and Liao [33] found that investors who need more help are less willing to accept AI-advising services. The authors speculate that the low transparency of AI-advising services may be the reason for this impact. Concerns about personal data security [35] and anxiety about healthcare [34] were also identified to induce users’ rejection of AI. Another possible explanation might be the concern about uniqueness neglect. For instance, Yalcin, Lim, Puntoni, and van Osselaer [31] showed that consumers respond less positively to an algorithmic decision maker, especially when the decision made by AI is favorable. An attribution process was proposed to explain this effect: consumers tend to deem a favorable decision made by a human as more reflective of their unique merits and, thus, feel themselves more deserving of the favorable decision. However, algorithmic decision makers usually rely on preset criteria. Thus, it is difficult to attribute the decision made by AI to one’s unique characteristics. Millet, Buehler, Du, and Kokkoris [37] identified that the perceived threats to human unique characteristics (i.e., artistic creativity) lead to responses against AI art generators. In a more direct investigation of the effects of uniqueness neglect, nine studies in Longoni, Bonezzi, and Morewedge [18] revealed consistent results showing that consumers tend to refuse AI medical applications for healthcare due to uniqueness neglect. Specifically, the authors provided evidence that people believe that AI is less able to identify their unique characteristics and circumstances in medical demands, which resulted in consumers’ reluctance to use AI medical services. Taken together, aversion to AI service providers may result from the concern about uniqueness neglect, the low perceived fit between AI and certain tasks, and the perceived inability of AI service providers. Uniqueness neglect, task fit, and algorithm performance are potential mechanisms for aversion to AI service providers.

Thirdly, most of the reviewed studies (49 papers) showed conditional results on users’ acceptance of AI service providers. The research further diverges into two streams. On one hand, some studies focused on exploring the influencing factors of AI service provider acceptance, and mainly employed the survey method. By far, the most attention was paid to perceived anthropomorphism of AI service providers, and related papers have consistently found a positive impact of anthropomorphism on users’ acceptance [48,50,51,96]. For example, Mishra, Shukla, and Sharma [48] showed that anthropomorphism has a positive impact on utilitarian attitude, which in turn increases acceptance of smart voice assistants. Pelau, Dabija, and Ene [51] revealed an indirect effect of anthropomorphism on the acceptance of AI devices, which is fully mediated by perceived empathy and interaction quality. Additionally, there are studies focused on the role of perceived transparency, accountability, and fairness. Shin, Kee, and Shin [41] conceptualized fairness, explainability, accountability, and transparency as key components of algorithm awareness, and found that higher levels of algorithm awareness increased users’ trust and self-disclosure to algorithmic platforms. Shin, Zhong, and Biocca [20] showed a positive relationship between AI service provider acceptance and users’ algorithmic experience, which was conceptualized as inherently related to fairness, transparency, and other components. Furthermore, various other factors were investigated in the reviewed studies, such as artificial autonomy [42], external locus of control [43], personalization [49], and user personality traits [50]. On the other hand, some studies focused on identifying the boundary conditions for AI service provider appreciation or aversion, and mainly adopted experimental methodologies. Related studies demonstrated that design characteristics [1,56,75,78,103], goal orientations [57], types of servicers’ responses [54,55], and assemblage of AI and humans [54,55,103] may significantly change users’ acceptant attitudes. For instance, Longoni and Cian [54] and Luo, Qin, Fang, and Qu [58] showed that users tend to be more accepting when AI is combined with humans. Tojib, Ho, Tsarenko, and Pentina [57] found that consumers with higher desire for achievement tend to accept service robots more. Taken together, users’ acceptant choices can be changed or even switched by technology-related characteristics, contextual factors, user personality traits, design features of AI application, and many other factors in AI service provider usage. Thus, although many efforts have been made to explore user acceptance of AI service providers and underlying mechanisms, more research is needed to identify key factors, clarify mixed findings, and conceptualize new constructs that provide unique understandings of AI service provider acceptance.

### 3.2. Results of Literature Review on User Acceptance of AI Task Substitutes

AI task substitutes are widely applied in the digital innovation of organizations. How physicians take advantages of AI diagnostic testing systems and how employees respond to substitutive decision-making AI systems have aroused researchers’ interest. In our analysis, 19 of the 80 reviewed papers focused on users’ acceptance of AI task substitutes (see Table 5). Most of the reviewed articles focused on the contexts of healthcare and future of work. Examples of AI task substitutes include AI-based autonomous tools, clinical decision support systems, AI-based hiring systems, etc. Regarding users’ acceptant responses to AI task substitutes, the reviewed studies also failed to reveal a consistent result. In the reviewed studies, 4 of the 19 papers reported aversion responses to AI task substitute acceptance, and 14 papers identified boundary conditions and antecedent factors for users’ acceptance. Surprisingly, there was only one article reporting a completely positive attitude toward AI task substitutes.

Firstly, four studies found that users tend to resist AI task substitutes in many contexts. In clinical diagnostic decision making, Liang and Xue [84] provided evidence from a longitudinal field survey that physicians expressed AI resistance due to the concern of face loss. The belief of professional autonomy and time pressure can even strengthen the resistant intentions. Strich, Mayer, and Fiedler [83] revealed that the feeling of professional identity threat may result in an employee’s reluctance to accept AI-based assistant systems. Apart from the concern about professional identity, factors related to AI usage barriers can also lead to negative responses to AI task substitutes. For example, Kim, Kim, Kwak, and Lee [22] found that employees may decline to use AI task substitutes because of the perception of technology overload (e.g., information overload, communication overload, and system feature overload). Taken together, users may not accept AI task substitutes because of the concerns that AI task substitutes may be difficult to use, reduce colleague interactions, produce unexplainable results, make employers or customers doubt their occupational competence, and even replace them in the workplace. The difficulty in AI usage, concerns about face loss, and feelings of threats to professional identity may drive the negative effect (i.e., users’ aversion to AI task substitutes). Furthermore, although these studies revealed an AI-aversion attitude, factors that may eliminate the negative effects are still worth exploring.

Secondly, 14 articles explored the boundaries and factors of AI task substitute acceptance, mainly in three contexts: medical AI, future of work, and human–robot interaction. In the context of medical AI, researchers mainly focused on why users (i.e., physicians) resist AI diagnostic systems and whether there are factors that can eliminate AI aversion. Results showed that users (i.e., physicians) tend to rely on their own judgements and resist AI task substitutes due to their self-esteem [84], self-expression of reputation and skill level [92], self-monitoring processes [87], and resistance to change and trust in AI [21]. However, monetary incentives and altruistic beliefs can eliminate the resistance to AI task substitutes [92], while for professional autonomy, time pressure will strengthen the AI-aversion response [84]. For example, Dai and Singh [92] distinguished experts into high-type and low-type. Based on game model, the authors found that low-type experts rely on AI advice more, while high-type experts tend to use their own diagnostic decision in order to distinguish themselves from low-type ones. In the context of future of work, various factors that determine user (i.e., employee and organization) acceptant attitudes toward AI in the workplace and teamwork have been investigated, including user perceptions of AI use (e.g., perceived threat, perceived restrictiveness, perceived autonomy) [21,84,91,94], user perception of AI (e.g., perceived capabilities of AI, performance expectancy) [21,90], and AI-enabled task/knowledge characteristics (e.g., skill variety, job complexity) [95]. For example, by interviewing senior managers, Hradecky, Kennell, Cai, and Davidson [88] revealed the key factors that influencing AI adoption in the event industry, including organizational technological practices, financial resources, the size of the organization, issues of data management and protection, and the risk of the COVID-19 pandemic. In the context of human–robot interaction, scholars focused on how users are willing to accept AI as competitors or collaborators. For example, Harris-Watson, Larson, Lauharatanahirun, DeChurch, and Contractor [98] suggested that perceived competence, compared with perceived warmth, was a more important decisive factor of users’ psychological acceptance of AI teammates. Dang and Liu [96] found that a malleable theory of the human mind increased users’ competitive responses to AI robots by reducing performance-avoidance goals, whereas it increased users’ cooperative responses to robots due to induced mastery goals. Hence, it can be seen that in different contexts of AI applications, users’ acceptance of AI task substitutes is influenced by different factors. Future research should identify the specificity of the studied context and the characteristics of human–AI interaction in order to explore the decisive factors of user acceptance behavior of AI based on specific contexts.

Finally, only one paper proved positive attitudes toward AI acceptance. Specifically, Zhang, Chong, Kotovsky, and Cagan [85] found that users tend to trust AI teammates more than human teammates. Furthermore, it worth noting that one paper explored whether customer acceptance or employee acceptance is more important for tourism practitioners in AI-related strategy development. Based on a field experiment, Fan, Gao, and Han [99] revealed the superiority of an imbalanced robotic strategy (i.e., focusing on customer acceptance more than employee acceptance) over a balanced one in service quality improvement, especially when customer demandingness is higher. As prior research focus on either users’ acceptance of AI service providers or users’ acceptance of AI task substitutes, this research integrated both perspectives and answered the question of how to balance the perceptions of two types of AI users, which provided a new research perspective for the acceptance of different AI roles.

## 4. Theoretical Perspectives Applied to User Acceptance of AI

Based on our observation, the theoretical frameworks most commonly used in the review articles are technology acceptance model (TAM) and the extended technology acceptance theories (e.g., the decomposed theory of planned behavior, unified theory of acceptance and use of technology). The TAM was proposed by Fred D. Davis in 1989 to explain user acceptance of computer technology [104]. The technology acceptance model is one of the most influential and robust theoretical models in the field of information technology acceptance research. In the TAM, perceived usefulness and perceived ease of use are two key factors, which both directly affect use attitude and indirectly affect use intention through use attitude. Moreover, perceived ease of use indirectly affects usage attitude through perceived usefulness. A large number of empirical studies has confirmed the TAM [105,106] and investigated external variables that have an impact on perceived usefulness and perceived ease of use [107,108]. In our analysis, five articles employed TAM as a theoretical framework [20,43,49,71,79] and explored antecedents of perceived usefulness and ease of use. Applying TAM to the AI service provider context, the papers supported the decisive role of perceived usefulness in promoting trust and behavioral intention to accept AI service providers, but found inconsistent results of the relationship between ease of use and acceptant attitude [20,49]. Necessary is further investigation into how TAM could be applied to the AI task substitute context, and how contextual factors influence the established relationships in TAM.

With the progress of technology and the deepening of research, the theoretical model of the technology acceptance model is constantly improved, and the explanatory power of the model is constantly enhanced. For instance, the theory of planned behavior (TPB) extended the TAM by separating usage attitude into three levels (i.e., subjective norm, perceived behavior control, and attitude toward the behavior) and specifying the antecedents (i.e., normative, control, and behavioral beliefs) for three attitude levels, respectively [109]. Furthermore, Taylor and Todd [110] proposed the decomposed theory of planned behavior (DTPB), which decomposed the normative, control, and behavioral beliefs in TPB into components. Specifically, the normative beliefs are decomposed into peers’ influence and superiors’ influence. The control beliefs are decomposed into self-efficacy, technology facilitating conditions, and resource facilitating conditions. The behavioral beliefs are decomposed into perceived usefulness, perceived ease of use, and compatibility. One of our reviewed articles adopted DTPB to examine how employees accept chatbots in an enterprise context. Results showed a strong influence of self-determination (attitude toward acceptance), but a weak impact of external ones (i.e., subjective norm and perceived behavior control).

Additionally, the unified theory of acceptance and use of technology (UTAUT) is an integrated theoretical framework of prior technology acceptance research [111]. In UTAUT, three decisive constructs (i.e., performance expectancy, effort expectancy, and social influence) are used to explain behavioral intention to use technology, while behavioral intention to use technology and facilitating conditions further affect technology use behavior. Furthermore, four moderators were also identified (i.e., age, gender, experience, and voluntariness of use). A variety of studies have empirically examined UTAUT and extended it in various contexts [112,113]. In our reviewed articles, Mamonov and Koufaris [53] applied UTAUT to explore users’ acceptance of AI service providers (i.e., smart thermostat) in a smart home context. The results revealed a weak effect of performance expectancy and an insignificant effect of effort expectancy on intention to adopt a smart thermostat. Meanwhile, techno-coolness, a novel factor proposed by the authors, has a stronger effect on users’ adoption intention. Similarly, Prakash and Das [21] tested UTAUT in clinical diagnostic context. Their study showed a consistent result with original UTAUT but an insignificant relationship between effort expectancy and users’ intentions to accept AI task substitutes (i.e., intelligent clinical diagnostic decision support systems). The authors explained that the ease of use may not be a decisive factor in the special context of clinical practice.

Taken together, TAM and the extended theories have the benefits of offering a comprehensive framework to investigate decisive factors of AI acceptance in specific contexts. However, there are also limitations. First, by adopting such models, most studies were restricted to survey methodologies. To deepen the understanding of users’ decision-making processes, more diverse methods should be integrated. Second, with the widespread application of information technology in various fields of society, the influencing factors of users’ intentions to accept new technologies may be different. As TAM and the extended models were first proposed about 20 years ago, these theories are worth extending in the era of artificial intelligence. Third, in our analysis, the established relationships between constructs in these models may not always be supported in different contexts. Further research may consider specific contextual factors influencing these relationships, conceptualize constructs for particular contexts, and make generalized theorizations.

### 4.1. Theoretical Perspectives Applied to User Acceptance of AI Service Providers

In terms of user acceptance of AI service providers, various theoretical perspectives have been identified. A total of 7 of the 61 articles adopted TAM and the extended theories, and 19 did not employ a specific theoretical framework. The remaining 36 articles identified 28 theories (see Table 4). The three most commonly used overarching frameworks are computers as social actors (CASA) theory (three papers), task–technology fit theory (three papers), and stimulus (S)–organism (O)–response (R) framework (three papers). Two theories were applied more than once, namely, social presence theory and attribution theory.

Social presence indicates a fairly generic sense of “being with others” during social interaction process [114]. When users experience AI service providers as actual social actors, they interact with AI service providers socially, and, thus, foster psychological and/or behavioral responses (e.g., perceive AI service providers as more credible, shift to a fairness evaluation, be more likely to self-disclose, and further increase intention to accept AI) [2,56]. In our analysis, social presence may serve as antecedent of usage attitude and/or intentions [2,48], but also be theorized as mediators that explain how human-like AI service providers are accepted [56]. This theory thereby serves as a theoretical foundation to explain how anthropomorphic features of AI service providers influence users’ acceptant intention. However, as this theory offers only a single construct of social presence, it is difficult to explain why the influences of different anthropomorphic features vary.

Attribution theory provides a theoretical foundation to understand users’ reactions to favorable and/unfavorable outcomes that are made by AI. According to attribution theory, people tend to infer the cause of events, and may attribute the causes to internal or external factors of the event. For example, researchers have found that people are inclined to attribute favorable events to themselves (e.g., the success was due to my hard work), while make external attribution to unfavorable events (e.g., the failure of exam was due to noise interference). In our review, researchers showed different mechanisms underlying users’ attribution on AI service providers. On one hand, users may attribute unfavorable events to contextual factors instead of AI service providers due to the belief that AI is stable and trackable [47]. On the other hand, users may attribute unfavorable events to AI service providers because they believe AI is given such high autonomy to hold responsibility to negative outcomes. Moreover, studies also found that there was no difference in users’ attribution on AI and human. The authors proposed one possible explanation that although AI may ignore uniqueness, humans may also not be objective [31]. Overall, this overarching theory provides a theoretical explanation for users’ behavioral responses to AI service providers, focusing on revealing the psychological process and influencing factors. Considering that people’s understanding of AI is complicated, the specificity of context and types of AI service provider should be fully considered when applying this theory to research.

### 4.2. Theoretical Perspectives Applied to User Acceptance of AI Task Substitutes

Among the 19 papers focusing on users’ acceptance of AI task substitutes, 2 used TAM and the extended theories as theoretical framework, and 9 did not explicitly adopt theoretical framework. The remaining eight articles adopted eight theories as theoretical frameworks particularly to explain users’ acceptance of AI task substitutes. Most of these theories were applied only once in our reviewed papers, including overarching frameworks (e.g., cognitive appraisal theory, the technology–organization–environment framework) and specific theories (e.g., the intergroup threat theory).

For example, cognitive appraisal theory offers an explanation on users’ coping mechanisms underlying their reaction to novel situations. According to this theory, people form initial appraisal of new situations by perception of the situation and their own knowledge. The coping mechanism then results from initial appraisal, and further results in different attitudes and behavioral intentions [115]. This theory, thus, provides an overarching framework for investigating how users react to a novel AI task substitute and/or new environment with an AI task substitute [90]. The technology–organization–environment framework is a fairly generic framework to understand the influence of technological, organizational, and environmental factors on organization’s acceptant decision making. However, it does not explicitly identify constructs that comprise the framework. Other theoretical models should be integrated to examine organizational acceptance of AI task substitutes in specific contexts. The intergroup threat theory is widely used to explain intergroup relations. Based on this theory, employees may experience threats from outgroup objects, named realistic threats and symbolic threats. Realistic threats refer to the risk of value loss, such as economic loss and threats to personal security, while symbolic threats are more concerned with the risk of identity loss, such as “uniqueness, self-identity, and self-esteem” [116]. This theory provides a narrow explanation for why users resist AI task substitutes from the relation threat perspective, which may help investigate how to alleviate users’ AI resistance.

Furthermore, dual process theory was utilized more than once in the reviewed studies. This theory identifies two modes of information processing, namely, heuristic process and systematic process. People’s attitudes, intentions, and behaviors rely on how they process information through the two processes [117,118,119]. Though heuristic processes, users tend to make evaluations unconsciously, instinctively, and intuitively, while through systematic processes, users rely more on cognitive, analytic, rational thinking to make decisions. This theory offers benefits for investigating users’ different reactions to AI task substitutes when different processes operate. For instance, Liang and Xue [84] suggested that physicians’ resistance to AI task substitutes is decreased when their systematic process (i.e., perceived usefulness of the AI system) is emphasized. Additionally, the research of Jussupow, Spohrer, Heinzl, and Gawlitza [87] provided evidence for how the two systems shift from one to the other dynamically by identifying the metacognition process.

## 5. Discussion

Overall, our review reveals inconsistent research findings on user attitudes and perceptions towards AI acceptance, as well as different factors and underlying mechanisms for AI service providers and AI task substitutes (see Table 4 and Table 5). For example, research on the superiority of AI over humans varies across different studies [30,34,85], users’ attribution of negative events is inconsistent [29,31,47], and the source of concern about AI seems to be influenced by the role of AI [36,37,83]. For AI service providers, users may appreciate the higher level of accuracy and reliability of AI applications, whereas they are concerned that AI cannot fit certain tasks due to uniqueness neglect and lack of affection. Trust and satisfaction with usage are main mechanisms for acceptance of AI service providers. For AI task substitutes, the main concern of users comes from professional identity threats and work performance after adopting AI. Nevertheless, factors that may eliminate the negative attitudes were explored. For instance, when users are incentivized by money [92], rationally evaluate the benefits of AI usage [91], or complete the identity adjustment in response to AI systems [83], the resistance towards AI task substitutes can be alleviated.

Extant research typically focuses on user perception, attitude, and acceptance behavior for specific AI applications, but few researchers have clarified the relationships between different AI roles and user perceptions, attitudes, and acceptance behavior. Furthermore, although research has explored many factors that can change users’ acceptant attitudes and behaviors towards AI applications, the underlying psychological processes are still worth investigating. Therefore, future research may further explore how the roles of AI help understand the inconsistencies in the reviewed studies. In addition, the following sections will provide three broad opinions on the limitations of the reviewed research, as well as guidelines for promoting future research on AI acceptance and decision-making mechanisms among users.

### 5.1. Key Findings and Future Research Directions

#### 5.1.1. Lack of Clarification of the Differences between Various AI Applications

Our analysis shows a lack of consistent definition and terminology of specific forms of AI application. As summarized in Table 4 and Table 5, the 80 reviewed studies identified 55 types of AI service providers and 19 types of AI task substitutes, totaling 71 types of AI applications, in about 25 kinds of contexts.

Although many researchers have demonstrated that the terminology of AI applications being studied is interchangeable with other terms, a considerable number of studies do not specifically explain the definition, characteristics, and human–machine interaction patterns in specific contexts of the AI applications being studied, nor do they conceptualize and theorize user acceptance based on these specific features. One of the consequences of this fragmentation is the poor generalizability of research conclusions and the inability to explain inconsistent results between different studies. For example, although research suggests that the definitions of chatbot and conversational AI are similar [120], previous studies have found mixed attitudes toward chatbot acceptance [2,32,61]. Without distinguishing the characteristics of the VA being studied from other VAs or pointing out the specificity of VA applications in the current context, it is difficult to fully discuss the inconsistent results mentioned above. This is also consistent with the “fragmented adhocracy” problem pointed out in previous review studies [26]. 

Furthermore, it may also result in the vague role of AI in usage. Due to the lack of AI application definition based on specific context and application type, most studies have failed to clarify the role played by AI applications in user usage, such as service providers and substitutes for employees. In fact, users will interact with artificial intelligence in different modes based on the role of the application. For example, when AI serves as an adviser, users will actively request AI to provide information for their decision-making; When AI serves as a service provider, users passively enjoy the services automatically provided by AI; When AI acts as a collaborator, users will work together with AI to complete tasks. Therefore, without a clear understanding of the role of AI in human-computer interaction, it is difficult to conduct in-depth research on users’ perception during the interaction process and their willingness to accept AI. 

Our review categorizes AI roles into two categories, namely AI service providers and AI task substitutes, and finds that users exhibit different acceptance attitudes and decision-making mechanisms when interacting with AI applications with different roles. For example, when AI serves as service providers, users’ AI resistance may stem from concerns about uniqueness neglect [18], task fit [39,77], and algorithm performance. While when AI serves as task substitutes, users tend to resist AI due to the difficulty in AI usage [22], concerns about face loss [84], and feelings of threats to professional identity [83]. Further research is recommended to provide empirical evidence for the differences in users’ acceptance of different AI roles, and to contribute unique knowledge on clear and accurate definitions, characteristics, and user interaction processes of different roles of AI applications.

#### 5.1.2. Limited Generalizability of Research Design

In the sample articles reviewed, the real behavioral data of organizations and individuals in the use of AI application have not been well exploited yet. The majority of our reviewed studies employed either surveys (32.10%) or behavioral experiments (35.80%) in a designed study setting. In contrast, only a few studies utilized field data (8.64%) or conducted multimethod research design (14.81%). Small sample sizes, controlled study settings in experimental research, imagined or recalled decision-making process, and limited perspective of individual users may restrict the generalizability of research conclusions in real-world settings. Moreover, as presented in the previous sections, the studies investigating AI acceptance have identified a pool of outcome variables, such as acceptance behavior [33,88,99], intention to use AI [42,82,91], and trust [77,85,97]. Yet, most studies in our sample measured user perceptions and behavioral intentions as outcome variables (83.95%). Only a few studies utilized users’ actual behavior in practice (16.05%). Although the extant research provided rich evidence in terms of AI acceptant intentions, we are still unclear as to whether these results still hold true in actual acceptant behaviors.

Thus, future research should seek opportunities to utilize real behavioral data for causality estimation, conduct field studies, combine multimethod research design, and consider the impact of individual and organizational characteristics on the acceptance of AI applications, to broaden the generalizability of research findings. In addition, considering the potential evolution of user attitudes toward new technologies during usage, we recommend longitudinal research in a field study setting for future studies to provide insights into the dynamic interactive process between users and AI applications and explore underlying mechanisms of AI acceptance.

#### 5.1.3. Conceptualization and Theorization in the Context of AI Acceptance

Many of the reviewed studies rely on the general technology acceptance models (e.g., technology acceptance model, unified theory of acceptance and use of technology, and health information technology acceptance model) as theoretical frameworks to explain users’ perceptions, attitudes, and acceptance behaviors towards AI applications [20,71,86]. This may ignore the possible changes in technology use behavior brought about by the massive application of information technology, which may affect the impact of key factors in the traditional technology acceptance models. For instance, Shin, Zhong, and Biocca [20] proved the significant impact of both perceived usefulness and ease of use on attitude toward algorithm services. Liu and Tao [49] found that perceived usefulness significantly affected trust and intention to use smart healthcare services, whereas perceived ease of use only predicted trust but did not influence usage intention directly. Lu, et al. [121] showed that perceived usefulness and ease of use were not associated with users’ acceptance of service robots. Additionally, the use of a new generation of AI applications can create new cognitive and affective experiences in human–computer interaction [20,53,64]. In the new era of AI, further research should rethink the boundaries of applying a single disciplinary theory to explain AI usage, make efforts in the extension of traditional models, and make new conceptualization of constructs in the specific context of AI acceptance.

Furthermore, in our analysis, quite a few studies did not identify the theoretical foundations (28 out of 80), or only used a generic overarching framework (e.g., stimulus (S)–organism (O)–response (R) framework; technology–organization–environment framework). Among those with specific theoretical frameworks, the reviewed studies for AI service providers mainly focused on particular responses (such as attribution and anthropomorphic perception), while in the few studies with explicit theoretical frameworks for AI task substitutes, the majority utilized psychological theories to explore the underlying mechanism of AI aversion. Overall, the reviewed research provided a theoretically fragmented picture of AI acceptance, but refrained from creating integrated theoretical frameworks in the specific context of AI acceptance. Future research should integrate theoretical insights of computer science, design science, psychology, and social science to enable more generalizable theorization for understanding AI acceptance.

### 5.2. Limitations

Due to the surge of AI-related research in recent years, this study only provided a review of relevant research in leading journals over the past three years. Although we made a deliberate effort to select leading journals with outstanding contributions to technology-acceptance-related knowledge, such as “Computers in Human Behavior”, which is highly regarded in the behavioral sciences, there is a possibility that relevant articles from other important journals may have been overlooked. To some extent, this review only conducted a descriptive review and statistics analysis of present research projects in leading journals. It is strongly recommended to conduct meta-analyses on a wider range of publications in the future to enhance our understanding of user acceptance of different roles of AI, as well as the impact of different factors on AI acceptance, and to analyze the research design and structure of related studies in specific contexts. Future research could benefit from including a broader range of journals to ensure a more comprehensive coverage of all relevant studies in the field.

## Figures and Tables

**Table 1:** Overview of reviewed studies.

Journal	Method	Number of Articles
Management Science	Empirical estimation	1
Marketing Science	Field experiment	1
	Game model	1
MIS Quarterly	Case study	1
Information Systems Research	Empirical estimation	1
	Field experiment	1
	Interview	1
	Survey	1
Journal of Marketing	Experiment	2
	Field experiment	1
	Mixed methods	1
Journal of Marketing Research	Experiment	1
	Field experiment	1
Journal of Consumer Research	Experiment	2
Journal of the Association for Information Systems	Case study	1
Journal of Management Information Systems	Experiment	3
	Mixed methods	1
International Journal of Information Management	Case study	1
	Interview	1
	Survey	8
	Mixed methods	3
Information & Management	Experiment	2
	Survey	2
	Mixed methods	1
Computers in Human Behavior	Experiment	19
	Field experiment	1
	Longitudinal study	1
	Survey	15
	Mixed methods	5

**Table 2:** Specifics of mixed methods.

Mixed Methods	Number of Articles
Qualitative methods and quantitative studies	6
Experiments and one survey	4
Empirical estimation on real-world data and 4 controlled experiments	1

**Table 3:** Overview of conceptualization of user acceptance of AI service providers.

Types of Outcome Variables		Number of Articles
Behavior	Acceptance behavior	5
	Usage behavior	6
	Purchase behavior	2
	User performance	1
Behavioral intention	AI resistance	3
	Intention to accept AI	18
	Intention to use AI	23
	Purchase intention	3
	Intention to self-disclosure	1
	User performance	4
Perception	Attitude	6
	Trust	14
	Satisfaction	6

**Table 4:** Overview of reviewed studies on user acceptance of AI service providers.

Source	Types of AI Service Provider	User Acceptance (or Not)		Theoretical Perspectives	Methods	Key Findings
		AI Appreciation	AI Aversion			
You, Yang and Li [17]	Judge–advisor system	√		Cognitive load theory.	Experiment	Individuals largely exhibit algorithm appreciation where they tend to adopt algorithmic advice to a greater extent. Related factors are also explored.
Gill [29]	Autonomous vehicle	√		Attribution theory.	Experiment	For negative events to pedestrians, people tend to consider autonomous vehicles more acceptable.
Schanke, Burtch and Ray [2]	Customer service chatbot	√		Social presence theory.	Field experiment	Consumers tend to be willing to self-disclose, shift to a fairness evaluation, and accept the offer provided by a human-like chatbot.
Peng, et al. [30]	AI service		√	Social cognition theory, task–technology fit theory.	Mixed method	Consumers tend to refuse AI for warmth-requiring tasks due to the low perceived fit between AI and task.
Longoni, Bonezzi and Morewedge [18]	AI medical application		√	Uniqueness neglect.	Experiment	With AI medical application, consumers are less likely to utilize healthcare, are less sensitive to differences in provider performance, exhibit lower reservation prices for healthcare, and derive negative utility.
Yalcin, et al. [31]	Algorithmic decision maker		√	Attribution theory.	Experiment	Consumers tend to response less positively to an algorithmic decision maker. Related factors are also explored.
Luo, et al. [32]	Chatbot		√		Field experiment	Although chatbots perform as effectively as proficient workers, the disclosure of chatbot identity will reduce customer purchase rates.
Ge, et al. [33]	AI financial-advising servicer		√		Empirical estimation	Investors who need more help are less likely to accept robot-advising services. Furthermore, the adjustment of adoption behavior based on recent robo-advisor performance may result in inferior performance of investors.
Park, et al. [34]	AI monitoring for healthcare		√		Experiment	Anxiety about healthcare monitoring and anxiety about health outcomes decreased the rejection of AI monitoring, whereas surveillance anxiety and delegation anxiety increased rejection. Meanwhile, individual-level risks and perceived controllability are significant moderators.
Aktan, et al. [35]	AI-based psychotherapy		√		Survey	Most participants reported more trust in human psychotherapists than in AI-based psychotherapists. However, AI-based psychotherapists may be beneficial due to the ability to comfortably talk about embarrassing experiences, having accessibility at any time, and accessing remote communication. Furthermore, gender and profession types may also affect choice of AI-based psychotherapists.
Formosa, et al. [36]	AI decision maker		√		Experiment	Users consistently view humans (vs. AI) as appropriate decision makers.
Millet, et al. [37]	AI art generator		√		Experiment	Users, especially among those with stronger anthropocentric creativity beliefs, perceived AI-made (vs. human-made) artwork as less creative and induced less awe, which led to less preference.
Drouin, et al. [38]	Emotionally responsive chatbot	Conditional			Experiment	In terms of negative emotions and conversational concerns, participants reported better responses to chatbot than human partners, whereas in terms of homophily, responsive chat, and liking of chat partner, participants showed better responses to human than chatbot.
Wang, et al. [39]	Shopper-facing technology	Conditional		Task–technology fit theory.	Survey	The authors identify three dimensions of shopper-facing technologies, named shopper-dominant (pre-) shopping technologies, shopper-dominant post-shopping technologies, and technology-dominant automations. Shoppers’ adoption intentions are determined by their evaluations on technology–task fitness.
Zhang, et al. [40]	Smart home service	Conditional		Surveillance theory.	Survey	The intention to use AI in a smart home context depends on the trade-offs of contextual personalization and privacy concerns.
Shin, et al. [41]	Algorithmic platform	Conditional		Privacy calculus theory.	Survey	The trust and self-disclosure to algorithms depend on users’ algorithm awareness, which depends on users’ perceived control of information flow.
Hu, et al. [42]	Intelligent assistant	Conditional		Mind perception theory.	Survey	Artificial autonomy of intelligent personal assistants is significantly related to users’ continuance usage intention, which is mediated by competence and warmth perception.
Canziani and MacSween [43]	Voice-activated smart home device	Conditional		Technology acceptance model.	Survey	Propensity for seeking referent persons’ opinions will increase perceived device utility. Perceived device utility and hedonic enjoyment of voice ordering are both positively related to consumers’ intentions to use the device for online ordering.
Sung, et al. [44]	AI-embedded mixed reality (MR)	Conditional		Stimulus (S)–organism (O)–response (R) framework.	Survey	The quality of AI, including speech recognition and synthesis via machine learning, significantly influences MR immersion, MR enjoyment, and perceptions of novel experiences, which collectively increase consumer engagement and behavioral responses (i.e., purchase intentions and intentions to share).
Wiesenberg and Tench [45]	Social robot	Conditional		Mediatization theory.	Survey	Leading communication professionals in Central and Western Europe as well as Scandinavia report higher concerns with ethical challenges of social bot usage, while professionals in Southern and Eastern Europe are less skeptical. In general, only a small minority of the sample reports readiness to use social bots for organizational strategic communication.
Song, et al. [46]	Intelligent assistant	Conditional		Theory of love.	Survey	AI application is able to promote users’ feeling of intimacy and passion. These feelings will positively impact users’ commitment, which further increase intention to use intelligent assistants.
Huo, et al. [47]	Medical AI	Conditional		Attribution theory.	Survey	Patients’ acceptance of medical AI for independent diagnosis and treatment is significantly related to their self-responsibility attribution, which is mediated by human–computer trust (HCT) and moderated by personality traits.
Mishra, et al. [48]	Smart voice assistant	Conditional		Flow theory and the theory of anthropomorphism.	Survey	Playfulness and escapism are significantly related to hedonic attitude, while anthropomorphism, visual appeal, and social presence are significantly related to utilitarian attitude. Smart voice assistant (SVA) usage is influenced more by utilitarian attitude than hedonic attitude.
Liu and Tao [49]	AI-based smart healthcare service	Conditional		Technology acceptance model.	Survey	Public acceptance of smart healthcare services is directly or indirectly determined by perceived usefulness, perceived ease of use, trust, and AI-specific characteristics.
Chuah, et al. [50]	Service robot	Conditional		Complexity theory.	Survey	Specific combinations of human-like, technology-like, and consumer features are able to increase intention to use service robots.
Pelau, et al. [51]	AI device	Conditional		The computers as social actors (CASA) theory.	Survey	Anthropomorphic characteristics of AI device indirectly influence acceptance and trust towards AI device through the mediation route of both perceived empathy and interaction quality.
Shin, Zhong and Biocca [20]	Algorithm system	Conditional		Technology acceptance model.	Mixed method	Users’ actual use of algorithm systems is significantly related to their algorithmic experience (AX).
Crolic, et al. [52]	Customer service chatbot	Conditional		Expectancy violation theory.	Mixed method	The effect of chatbot anthropomorphism on customer satisfaction, overall firm evaluation, and subsequent purchase intentions depends on customers’ emotional state. An angry emotional state leads to a negative effect.
Mamonov and Koufaris [53]	Smart thermostat	Conditional		Unified theory of acceptance and use of technology.	Mixed method	The smart thermostat adoption intention is mainly determined by techno-coolness, less by performance expectancy, and not by effort expectancy.
Longoni and Cian [54]	AI-based recommender	Conditional			Experiment	Consumers are more likely to adopt AI recommendations in the utilitarian realm, while they tend to adopt the, less in the hedonic realm. Related factors are also explored.
Lv, et al. [55]	AI service	Conditional		Social response theory.	Experiment	In service recovery, a high-empathy AI response can significantly increase customers’ continuous usage intention.
Garvey, Kim and Duhachek [19]	AI marketing agent	Conditional		Expectations discrepancy theory.	Experiment	Consumers tend to react positively (i.e., increased purchase likelihood and satisfaction) to AI agent for bad news, while they react negatively to good news offered by AI agent.
Al-Natour, Benbasat and Cenfetelli [1]	Virtual advisor	Conditional		Social exchange theory.	Experiment	The perceptions of a virtual advisor and the relationship with a virtual advisor are both determinants in self-disclosure intention.
Lv, Yang, Qin, Cao and Xu [55]	AI music generator	Conditional		Role theory.	Experiment	The acceptance of an AI music generator as a musician is significantly related to its humanlike traits, but not influenced by its autonomy to create songs.
Kim, et al. [56]	AI instructor	Conditional		Social presence theory.	Experiment	An AI instructor with a humanlike voice (vs. with a machinelike voice) improves students’ perceived social presence and credibility, which further increases intention to enroll in AI-instructor-based online courses.
Tojib, et al. [57]	Service robot	Conditional			Experiment	Service robot adoption is directly or indirectly determined by desire for achievement (PAP), desire to avoid failure (PAV), spontaneous social influence, and challenge appraisal.
Luo, et al. [58]	AI coach	Conditional		Information processing theory.	Field experiment	Middle-ranked human agents benefit more from the help of an AI coach, while both bottom- and top-ranked agents show limited incremental gains, because bottom-ranked agents exhibit information overload problem and top-ranked agents hold the strongest aversion to an AI coach.
Ko, et al. [59]	AI-powered learning app	Conditional		Temporal construal theory.	Empirical estimation	Students living in the epicenter of the COVID-19 outbreak (vs. those do not) tended to use AI-powered learning app less at first, but increased, regularized their usage, and rebounded to a curriculum path with time.
Luo, et al. [60]	Emotion-regulatory chatbot	Conditional		Interpersonal emotion management (IEM) theory.	Experiment	Perceived interpersonal emotion management strategies significantly affected positive word-of-mouth, which was sequentially mediated by appraisals and post-recovery emotions.
Chandra, et al. [61]	Conversational AI agent	Conditional		Media naturalness theory.	Mixed method	Human-like interactional (i.e., cognitive, relational, and emotional) competencies in conversational AI agents increased user trust and further improved user engagement with the agents.
Chi, et al. [62]	AI service robot	Conditional		Artificially Intelligent Device Use Acceptance (AIDUA) framework.	Survey	Trust in AI robot interaction affected use intention. Uncertainty avoidance, long-term orientation, and power distance were significant moderators.
Chong, et al. [63]	AI advisor	Conditional			Mixed method	The choice to accept or reject AI suggestions was determined by human self-confidence rather than confidence in AI.
Rhim, et al. [64]	Survey chatbot	Conditional			Experiment	Humanization applied survey chatbot (vs. baselinebot) is perceived as more positive, with higher anthropomorphism and social presence. Participants spent more time in HASbot interaction and indicated higher levels of self-disclosure, satisfaction, and social desirability bias with HASbot than with baselinebot.
Hu, et al. [65]	AI assistant	Conditional			Longitudinal study	Users perceived less risk and were more willing to use AI assistants in shopping when perceived power fits desire for power.
Benke, et al. [66]	Emotion-aware chatbot	Conditional			Experiment	Control levels induced users’ perceptions of autonomy and trust in emotion-aware chatbots, but did not increase cognitive effort.
Plaks, et al. [67]	Robot	Conditional			Experiment	The authors varied the robotic counterpart’s humanness by displaying values and self-aware emotions from low to high levels. As values varied from low to high levels, participants tended to choose the cooperative option; whereas as levels of self-aware emotions increased, participants were more likely to choose the competitive option. Trust was identified as key mechanism.
Jiang, et al. [68]	AI-powered chatbot	Conditional		Social exchange theory and resource exchange theory.	Survey	Responsiveness and a conversational tone sequentially increased customers’ satisfaction with chatbot services, social media engagement, purchase intention, and price premium.
Munnukka, et al. [69]	Virtual service assistant	Conditional		Computers as social actors (CASA) theory.	Experiment	The interaction with a virtual service assistant (i.e., perceived anthropomorphism, social presence, dialog length, and attitudes) increased recommendation quality perceptions and further improved trust in VSA-based recommendations.
Chua, et al. [70]	AI-based recommendation	Conditional			Experiment	Attitude toward AI was positively related to behavioral intention to accept AI-based recommendations, trust in AI, and perceived accuracy of AI. Uncertainty level was a significant moderator.
Yi-No Kang, et al. [71]	AI-assisted medical consultation	Conditional		Health information technology acceptance model.	Survey	Three dimensions of health literacy were identified as healthcare, disease prevention, and health promotion. Disease prevention was significantly associated with attitudes toward AI-assisted medical consultations through mediation of distrust of AI, whereas health promotion was also positively related to attitudes toward AI-assisted medical consultations through mediation of efficiency of AI. Furthermore, digital literacy was associated with attitudes toward AI-assisted medical consultations and mediated by both distrust and efficiency of AI.
Liu, et al. [72]	Task-oriented chatbot	Conditional		D&M information system success model.	Mixed method	Relevance, completeness, pleasure, and assurance in both mainland China and Hong Kong sequentially increased satisfaction and usage intention. Privacy concerns in both regions did not significantly affect satisfaction. Response time and empathy were significantly associated with satisfaction only in mainland China.
Wu, et al. [73]	Shared autonomous vehicle	Conditional		Trust-in-automation three-factor model.	Survey	Anthropomorphism negatively influenced human–SAV (i.e., shared autonomous vehicle) interaction quality when participants were male, with low income, low education, or no vehicle ownership.
Hu, et al. [74]	Conversational AI	Conditional		Interaction of person-affect-cognition-execution (I-PACE) model.	Survey	Social anxiety increased problematic use of conversational AI, which was mediated by loneliness and rumination. Mind perception was a significant moderator.
Alimamy and Kuhail [75]	Intelligent virtual assistant	Conditional		Human–computer interaction theory and stimulus–organism–response theory.	Survey	Perceived authenticity and personalization increased commitment, trust, and reusage intentions, which were mediated by user involvement and connection.
Im, et al. [76]	Voice assistant	Conditional		Computers as social actors (CASA) theory.	Experiment	When users engaged in functional tasks, voice assistants with a synthetic voice increased perceived fluency, competence perception, and attitudes.
Jiang, et al. [77]	Chatbot	Conditional		Task–technology fit theory.	Mixed method	Conversational cues were associated with human trust, which was mediated by perceived task-solving competence and social presence. The extent of users’ ambiguity tolerance and task creation were significant moderators.
Dubé, et al. [78]	Sex robots	Conditional			Survey	Correlational analyses showed that willingness to engage with and perceived appropriateness of using sex robots were more closely related to erotophilia and sexual sensation seeking than any other traits. Mixed repeated-measure ANOVAs and independent sample t-tests with Bonferroni corrections also showed that cismen and nonbinary/gender-nonconforming individuals were more willing to engage with sex robots and perceived their use as more appropriate than ciswomen.
Wald, et al. [79]	Virtual assistant	Conditional		Technology acceptance model, uses and gratifications theory, and the first proposition of the differential susceptibility to media effects model.	Survey	Hedonic motivation was the key factor influencing parents’ willingness to co-use the virtual assistant with their child(ren).
Pal, et al. [80]	Conversational AI	Conditional		Stimulus organism response framework and theory of love.	Mixed method	Love (i.e., passion, intimacy, and commitment) significantly influenced the usage scenario. The agent personality was a significant moderator.
Oleksy, et al. [81]	Algorithms in urban governance	Conditional			Mixed method	Lower level of perceived friendliness of the city increased users’ reluctance to accept algorithmic governance. Cooperation between algorithms and humans increased acceptance of algorithms, perceived friendliness, and controllability of the city.
Lee, et al. [82]	AI-embedded system	Conditional		HAII-TIME (Human–AI Interaction from the perspective of the Theory of Interactive Media).	Experiment	Users tended to view the system with explicit or implicit machine-learning cues as a helper and trusted it more.

**Table 5:** Overview of reviewed studies on user acceptance of AI task substitutes.

Source	Types of AI Task Substitute	User Acceptance (or Not)		Theoretical Perspectives	Method	Key Findings
		AI Appreciation	AI Aversion			
Strich, et al. [83]	Substitutive Decision-Making AI System		√	Professional role identity	Case study	The introduction of the Substitutive Decision-Making AI System makes employees feel their professional identities are threatened; thus, they strengthen and protect their professional role identities.
Liang and Xue [84]	Clinical decision support system		√	Dual process theory	Survey	Physicians may resist clinical decision support system (CDSS) recommendations. Related factors are also explored.
Kim, Kim, Kwak and Lee [22]	AI assistant		√		Field experiment	Although AI-generated advices lead to better service performance, some employees may not utilize AI assistance (i.e., AI aversion) due to unforeseen barriers to usage (i.e., technology overload).
Zhang, et al. [85]	AI teammate	√			Experiment	Compared with human teammates, users trust AI teammates more, accepting the AI’s decisions.
Brachten, et al. [86]	Enterprise bot	Conditional		The decomposed theory of planned behavior	Survey	Both intrinsic and external motivations of employees positively influence the intention to use Enterprise Bots, and the influence of intrinsic motivation is stronger.
Jussupow, et al. [87]	AI-based system	Conditional		Dual process theory	Interview	Physicians tend to use metacognitions to assess AI advice, and the metacognitions determine whether physicians make decisions based on AI or not.
Hradecky, et al. [88]	AI application	Conditional		The technology—organization—environment framework	Interview	The degree of confidence in organizational technological practices, financial resources, the size of the organization, issues of data management and protection, and the COVID-19 pandemic determine the adoption of AI in the event industry.
Vaast and Pinsonneault [89]	Digital technology	Conditional			Case study	AI adoption relies on the constant adjustment and redefinition of people’s occupational identity.
Chiu, et al. [90]	Enterprise AI	Conditional		Cognitive appraisal theory	Survey	Perceptions of AI’s operational and cognitive capabilities significantly increase affective and cognitive attitudes toward AI, while concerns regarding AI significantly decrease affective attitude toward AI.
Prakash and Das [21]	Intelligent clinical diagnostic decision support system	Conditional		Unified theory of acceptance and use of technology	Mixed method	Performance expectancy, effort expectancy, social influence, initial trust, and resistance to change are significantly related to intention to use.
Yu, et al. [91]	Technological agency	Conditional			Experiment	Control and restrictiveness significantly affect users’ perceived relation with technological agents and acceptance.
Dai and Singh [92]	AI diagnostic testing	Conditional		Game theory	Game model	High-type experts tend to use their own diagnostic decision, while low-type experts rely on AI advice more. Related factors have also been explored.
Gkinko and Elbanna [93]	AI chatbot	Conditional			Case study	The dominant mode of interaction and the understanding of the AI chatbot technology significantly contribute to users’ appropriation of AI chatbots.
Ulfert, et al. [94]	Agent-based decision support system (DSS)	Conditional			Experiment	High DSS autonomy increased users’ information load reduction and technostress, but decreased user intention. Job experience strengthened the impact on information load reduction, but weakened the negative effect on user intention.
Verma and Singh [95]	AI-enabled system	Conditional		Prospect theory, job design theory	Survey	AI-enabled task characteristics (job autonomy and skill variety) and knowledge characteristics (job complexity, specialization, and information processing) are significantly related with innovative work behavior. Meanwhile, perceived substitution crisis is a significant moderator.
Dang and Liu [96]	AI robot	Conditional			Experiment	A malleable theory of the human mind negatively affected performance-avoidance goals, and further positively affected competitive responses to robots. Meanwhile, a malleable theory of the human mind positively affected mastery goals, and further positively affected cooperative responses to robots. Further, Chinese participants were less competitive and had more cooperative responses to AI robots than British participants.
Westphal, et al. [97]	Human–AI collaboration system	Conditional		Cognitive load theory	Experiment	Decision control was positively associated with user trust, understanding, and user compliance with system recommendations. Providing explanations may not only reenact the system’s reasoning, but also increase task complexity; the effectiveness relies on the user’s cognitive ability in complex tasks.
Harris-Watson, et al. [98]	AI teammate	Conditional		Tripartite model of human newcomer receptivity	Experiment	Perceived warmth and competence affect psychological acceptance, and further positively impact perceived HAT viability.
Fan, et al. [99]	AI robot	Conditional		Information processing theory	Field experiment	An imbalanced robotic strategy is superior to a balanced one for service quality. In addition, when customer demanding is high, a customer-focused robotic strategy (i.e., higher customer acceptance of robots than employee acceptance) is the optimal choice to improve service quality. However, when frontline task ambidexterity is high, the positive effects of imbalanced robotic strategy on service quality diminish.

## Data Availability

Not applicable.
